# Use of the long-term quality of life assessment in the decision to indicate surgery in patients with chronic rhinosinusitis^☆^

**DOI:** 10.1016/j.bjorl.2018.03.011

**Published:** 2018-04-22

**Authors:** Pablo Pinillos Marambaia, Manuela Garcia Lima, Hélder Macário, Amaury de Machado Gomes, Leonado Marques Gomes, Melina Pinillos Marambaia, Otávio Marambaia dos Santos

**Affiliations:** a Instituto de Otorrinolaringologia Otorrinos Associados (INOOA), Salvador, BA, Brazil; b Escola Bahiana de Medicina e Saúde Pública, Salvador, BA, Brazil

**Keywords:** Rhinosinusitis, Quality of life, Surgery, Rinossinusite, Qualidade de vida, Cirurgia

## Abstract

**Introduction:**

Quality-of-life questionnaires have been used to support decision-making in patients with chronic rhinosinusitis in the past decade. The choice of treatment in practice, however, also considers the patient's decision.

**Objective:**

To assess the long-term quality of life of patients with chronic rhinosinusitis who decided to avoid surgery.

**Methods:**

This is a prospective longitudinal study with a group of patients with chronic rhinosinusitis, with and without indication for surgery, with application of the questionnaire SNOT-22 in two periods: between 2011 and 2012 and between June and August 2016, via email.

**Results:**

Data were collected from 42 patients, of which 13 presented indications for surgery and 29 were not indicated for surgery. The average quality of life score was 42.1 (±16.4) in the group with an indication for surgery and 40.6 (±23.4) in the group without this indication, *p* = 0.84. All the patients were assessed by a single doctor with blinding in relation to the initial score. No differences were detected between the groups. The impact of the chronic rhinosinusitis was reduced even among the patients with the indication for surgery. Both groups scored over 40.

**Conclusion:**

This study can help predict the impact of the chronic rhinosinusitis over time and better adjust expectations with non-surgical treatment.

## Resumo

**Introdução:**

Questionários de qualidade de vida têm sido usados na última década para apoiar a tomada de decisão em pacientes com rinossinusite crônica. Entretanto, na prática, a escolha do tratamento também considera a decisão do paciente.

**Objetivo:**

Avaliar a qualidade de vida em longo prazo de pacientes com rinossinusite crônica que decidiram não se submeter à cirurgia.

**Método:**

Estudo longitudinal prospectivo com um grupo de pacientes com rinossinusite crônica, com e sem recomendação de cirurgia, com aplicação do questionário *Sino-Nasal Outcome Test* 22 (SNOT-22, teste de desfecho sinonasal) em dois períodos: entre 2011 e 2012 e entre junho e agosto de 2016, via e-mail.

**Resultados:**

Foram coletados dados de 42 pacientes, dos quais 13 tiveram recomendação para cirurgia e 29 não tiveram. O escore médio de qualidade de vida foi de 42,1 (± 16,4) no grupo com indicação de cirurgia e de 40,6 (± 23,4) no grupo sem essa indicação, *p* = 0,84. Todos os pacientes foram avaliados por um único médico, cegado para o escore inicial. Não foram detectadas diferenças entre os grupos. O impacto da rinossinusite crônica foi reduzido mesmo entre os pacientes com indicação de cirurgia. Ambos os grupos apresentaram um escore acima de 40.

**Conclusão:**

Este estudo pode ajudar a prever o impacto da rinossinusite crônica ao longo do tempo e ajustar de forma mais adequada as expectativas com o tratamento não cirúrgico.

## Introduction

Classically, the treatments offered to patients with chronic rhinosinusitis (CRS) are medical and, when this treatment fails, surgery.^1^ In practice, the decision of which treatment to indicate is not as Cartesian and does not follow a well-defined criterion. The decision is often multifactorial and depends on patient factors, such as a sense of risk, cultural factors, the cost of treatment, and the physician.^2^ In recent reports, some authors demonstrated that, according to the patients, the most important factors in the choice of treatment are the impact that CRS has on their physical and mental well-being, the reduction in their quality of life (QOL), the loss of productivity, and the cost of treatment.^3^

The assessment of the QOL of patients has been used frequently in a range of different diseases and it is no different in the follow-up of patients with CRS.^4^ Disease-specific questionnaires, initially administered to assess disease over time or assess the impact of interventions in the same group of patients, have always been used in the hope of supporting the standardisation of conducts to prevent unnecessary procedures and improve care for this population.

The Sinonasal Outcome Test 22 (SNOT-22) is an easily applied questionnaire that has been validated for use in Portuguese.^5^ This instrument contains 22 questions about symptoms that are possibly related to chronic rhinosinusitis. Each symptom is given a score from 0 (zero) to 5 (five), where zero indicates the absence of a problem and five indicates the worst possible problem. Therefore, the higher the score, the worse the quality of life of the subject.^5^, ^6^ According to the European Position Paper on Rhinosinusitis and Nasal Polyps (EPOS) 2012,^1^ the SNOT-22 is a good tool for assessing QOL in patients with CRS because it can be used repeatedly and represented in graphs (SNOT gramas) containing the SNOT-22 scores in several moments in time. These graphs clearly display the result of medicinal and surgical interventions and complications over time.

Several studies using the SNOT-22 show that patients with higher scores, that is, those who have been most affected by the disease and have the worst QOL, clearly improve with surgical treatment in comparison with sufferers who undergo continued drug therapy. However, low scores do not seem to determine the choice of treatment.^7^ Other data, however, show no differences between surgical treatment and continued drug therapy for certain groups.^8^ The use of questionnaires could help stratify patients with the greatest likelihood of surgery, for example, and, consequently facilitate or reinforce an indication for referral to the ENT. Questionnaires could also be used by non-specialist physicians in primary care.

The methodology employed in the various works generally requires separate groups for every QOL score. Patients are typically divided by choice or randomisation in groups for the maintenance of drug therapy or surgical intervention. The personal choice factor is not usually taken into account in clinical studies, but it has a huge influence on the everyday practice. The aim of this paper is to compare the quality of life of patients with CRS who decided not to undergo surgery even when surgery was recommended by the ENT, and a group of patients who did not receive an indication for surgery, after 4 years.

## Methods

This is a longitudinal, prospective, and observational study, in which patients with CRS were monitored for at least 4 years, from the initial consultation in 2011 and from 2012 to 2016.

All the patients were assessed in the first ENT consultation. After confirmation of CRS (according to the clinical criteria of the EPOS-2012), they completed a registration form with demographic data and the SNOT-22 questionnaire, and signed an informed consent statement.

The diagnosis of chronic rhinosinusitis was determined using the clinical criteria of the EPOS-2012, whereby chronic rhinosinusitis is defined by the presence of two or more symptoms of nasal obstruction/congestion/blockage, anterior or posterior rhinorrhea, hyposmia/anosmia, and facial pain/pressure for 12 weeks or more. One of these symptoms had to be nasal obstruction/congestion/blockage or anterior or posterior purulent rhinorrhea.^1^

The inclusion criteria were literate patients with chronic rhinosinusitis and patients over 18 years of age.

The criteria for exclusion were illiterate patients, smokers, patients with immune deficiency, cystic fibrosis or primary ciliary dyskinesia, patients with benign or malignant nasal tumours, patients with granulomatous diseases and vasculitis, patients who had previously undergone surgery and subjects who refused to participate in the study.

During the analysis period, the subjects were divided into 2 groups: one group, called the surgical indication group, had received a medical indication for surgical treatment, but chose not to undergo the procedure. The other group, called the control group or the clinical treatment group, did not receive the indication of surgery, that is, they continued with the clinical treatment.

Surgery was indicated when maximum clinical treatment had failed for at least 6 (six) weeks. Maximum clinical treatment refers to the use of systemic or topical corticosteroids, antibiotics, and nasal saline wash.

The failure of clinical treatment was defined when the patient stated that the symptoms had not improved. In the case of lack of response, a computed tomography scan was requested to evaluate the condition, followed by the possible scheduling of future surgery.

Surgery was also indicated when tomographic analysis led to the diagnosis of a condition that required surgical treatment, namely significant anatomical changes such as obstructive septum deviation, large or obstructive middle turbinate pneumatisation or extensive sinonasal polyposis, and rhinosinusitis of dental or fungal origin.

The indication for surgery was based on the criteria mentioned above and on the conduct of a single ENT professional who was blinded regarding the initial SNOT-22 score of the patients.

Between June and August of 2016, the patients were contacted by telephone and, later, by email. During this contact, they were invited to participate in this study, complete the SNOT-22 questionnaire, and return it to the researcher. In addition to the questionnaire, the participants signed a new informed consent statement.

The study in question was approved by the ethics committee of the Escola Bahiana de Medicina and registered in Platform Brasil under number 54870816.1.0000.5544.

A sample was calculated using WinPepis software version 11.62, where we used the standard deviation of the SNOT-22 score of Smith et al.^7^ involving the comparison of scores from operated patients versus non-operated patients (SD = 19.1 and 22.1, respectively), and detected a difference of 25 points. In this case, 22 patients were needed, and divided into two groups of 11 subjects. Consequently, our sample exceeds the required number of participants.

The results were tabulated and analysed using SPSS-17 software.

The categorical demographic data like gender and presence of comorbidities and allergies were presented using the valid percentile. The Chi-square test was used to compare the categorical variables between the groups.

The score of the SNOT-22 questionnaire was described using the average and standard deviation since the sample distribution was normal.

The averages between the groups were compared using the unpaired *t*-test.

The unpaired *t*-test was also used to compare the average score of each item of the SNOT-22 individually.

The alpha error was considered acceptable when *p* < 0.05.

## Results

A total of 42 patient records were analysed, of which 13 were patients with an indication for surgery and 29 with an indication for drug therapy. Table 1 shows the demographic characteristics of the sample.

**Table 1:** Sociodemographic characteristics of the sample of patients with chronic rhinosinusitis and a surgical indication (indication group) and of patients with chronic rhinosinusitis with an indication for clinical treatment (clinical group).

Variables	Surgical indication group (*n* = 13)	Clinical treatment group (*n* = 29)	Significance (*p*)
*Gender (%)*			
Male	06 (46.2)	12 (41.4)	0.517
Female	07 (53.8)	17 (58.6)	

*Age (years)*	43.5 ± 3.1	38.4 ± 2.5	0.438
*Co-morbidities*			
SAH	02	02	0.81
DM	0	02	0.307
Asthma	03	02	0.167
Others	0	02	0.307

*Allergy to medication (%)*			
Yes	04 (30.8)	07 (24.1)	0.736
No	09 (69.2)	22 (75.9)	

*Respiratory allergy (%)*			
Yes	01 (7.7)	02 (6.9)	0.793
No	12 (92.3)	27 (93.1)	

Surgical indication group, patients referred for surgery; clinical treatment group, patients referred for clinical treatment.

Significance level *p* < 0.05.

With regard to the SNOT-22 score after 4 years of monitoring, we found that the group that evolved to surgical treatment scored 42.1 ± 16.4 and the clinical treatment group averaged 40.6 ± 23.4 (Table 2).

**Table 2:** Quality of life score with the SNOT-22 of the groups after 4 years.

Variable	Surgical indication group	Clinical treatment group	Significance (*p*)
SNOT-22	42.1 (±16.4)	40.6 (±23.4)	0.84

SNOT-22, Sino Nasal Outcome Test. Unpaired *t*-test. Average (standard deviation).

Significance level *p* < 0.05.

**Figure 1:**
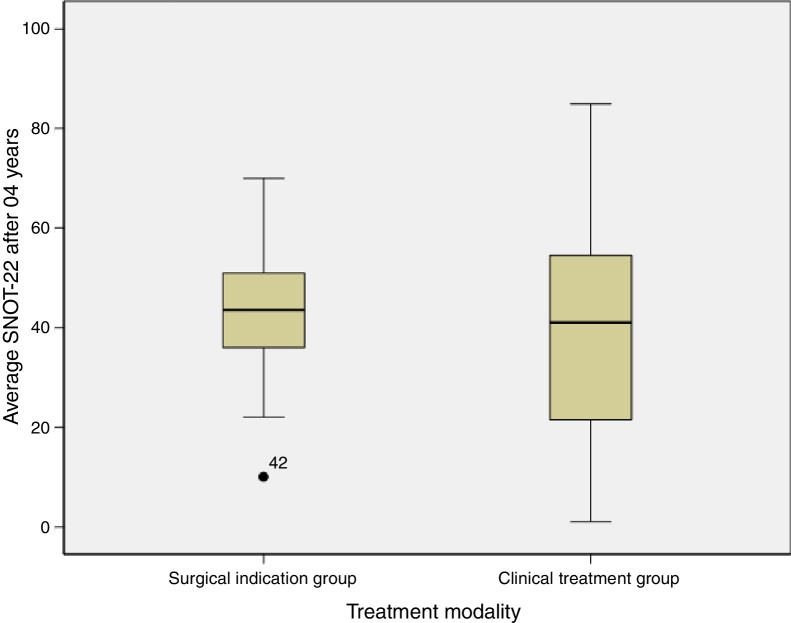
Comparison of the SNOT-22 score averages of the groups after 04 years.

Fig. 1 shows the comparison of the SNOT-22 score averages of the groups after 04 years.

The comparison of each item (symptom) in the SNOT-22 questionnaire and the subdomains of the SNOT-22 separately did not differ between the groups.

**Figure 2:**
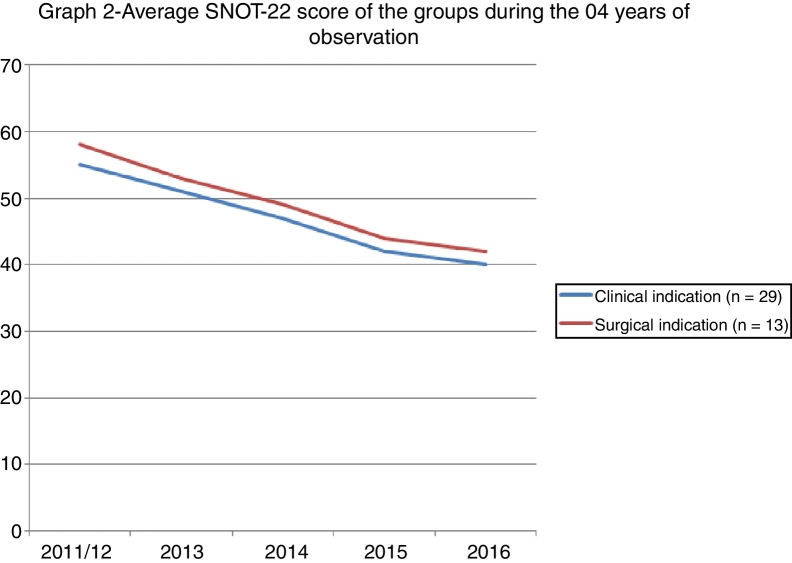
The evolution of the scores of the two groups after 04 years of observation.

Fig. 2 shows the evolution of the scores of the two groups after 04 years of observation.

## Discussion

Drug therapy is the standard treatment of patients with uncomplicated chronic rhinosinusitis. Most highly qualified otolaryngologists would not recommend surgery, knowing that a large number of patients with uncomplicated CSR may show improvements or stabilise the disease with drug therapy.^9^ Surgery is indicated when clinical treatment fails or in the case of complications.^10^ Endoscopic sinus surgery (ESS) is the surgical modality indicated in these cases and has shown good results since the 1990s.^10^

The choice of treatment and the timing of surgery are not simple and do not obey a clearly defined rule. The bias of patients, such as cultural factors, cost of treatment, aversion to risk, and the doctor-patient relationship, are unknown and underexplored.^2^ It is believed however, that QOL assessment instruments can significantly support this decision.^11^ Data on the exact weight of these instruments in the change of conduct still allow some room for discussion. Logically, data that show the benefits of surgery rather than drug therapy can help explain and support patients in making the best decision.

In this study, we assessed a group of patients who decided not to undergo surgery, although surgery had been indicated. The indication of surgery observes the rule of previous studies that reserve this option for patients who do not show improvements with the so-called maximum clinical treatment. The QOL comparison between the group with indication for surgery and the group without indication for surgery showed no statistical difference after 4 years of follow-up (*p* = 0.84).

The evaluation of the QOL after 4 years showed a reduction in the SNOT-22 score of all the patients; however, there was no difference between the groups. The pathophysiological rationale is that patients with an indication for surgery should have worse scores or a gradual reduction of the scores over time. This result can strengthen the indication for surgery or help predict the evolution of a surgical patient. Our data says otherwise. Both groups evolved towards a reduction of the scores, and no discrepancy was found between the groups. These data can translate a simple statistical phenomenon of regression towards the mean after the first measurement, that is, they can show that the first measurement of a sequence may have been overrated and that the subsequent measurements merely portray the reality of the data. Serial samples would be needed to rule out this hypothesis. Another hypothesis refers to the measurement bias since the first measurement was made during a medical consultation, that is, the patient probably sought the service because he or she was symptomatic and responded to the questionnaire in this opportunity. The second measurement was performed remotely, i.e. without face-to-face contact, which may have portrayed patients outside the symptomatic period or between episodes. This occurred in both groups, which may have minimised the occurrence of bias.

This result leads us to conclude that the fact that a patient does not undergo surgery does not worsen the illness or its impact on the QOL of the patient; on the contrary, the disease may even “improve”. However, when we look at the average final score of the SNOT-22, we see that both groups still had high scores – Surgical indication group: 42.1 (±16.4) versus the Clinical treatment group: 40.6 (±23.4). In the case of surgery, the score may have significantly dropped. Steele et al.^12^ maintain that patients with a low score (SNOT-22 < 30) report a stable QOL with drug therapy, and that when these patients undergo surgery, they experience a more sensitive clinical change

The literature provides abundant data to support surgical treatment. Although the authors did not defend the use of questionnaires as a standalone decision-making tool, they believe these mechanisms provide important support for counselling and risk stratification. Hopkins et al.,^3^ in a study with the English population, suggest that patients with SNOT-22 scores above 30 are more likely to show a clinical improvement after surgery than patients with scores below 20, in which case surgery is not encouraged. Rudmik et al.^13^ studied the American population and found similar results. In this respect, it is important to remember that even patients without chronic rhinosinusitis score around 7 or 8 in the SNOT-22.^14^ This means that patients with low scores or fairly low scores do not have margin for improvement and should be analysed carefully since their scores are very similar to those of people without the disease.

The cost of treatment should also be taken into account. In this study, the cost of medication was not assessed over the studied period. Depending on the reality of study location, this can be an important factor for deciding which treatment to indicate. Smith et al.^7^ found that the QOL improved in the surgical and non-surgical groups, although the latter group received more antibiotics, prescription nasal sprays, and sinus medications in tablet form than the former group.

In this study, the subdomains of the SNOT-22 were analysed separately and showed no differences between the two groups. The subdomains of nasal, extra-nasal, and aural/facial symptoms, sleep disorders and psychological symptoms are part of the instrument and help to assess various aspects of patients and, subsequently, evaluate the impact of CSR on their QOL.^15^ Levy et al.^16^ show in patients with a low SNOT-22 score that, while the total score may not differ, when analysed separately, the subdomains may have discrepancies between the various groups and consequently influence the choice of treatment. The scores of nasal, extra-nasal, and aural/facial symptoms seem to be determining factors. Curiously, a previous study showed that the domains of psychological symptoms and sleep disturbances were more closely associated with the decision to undergo surgery.^11^

Using the SNOT-22 to punctually assess patients does not seem to be the most reliable method in decision making. Serial assessments can help trace the real evolution of each patient and detect periods in which the symptoms or the disease get worse. Snot-grams are graphs that are made with different measurements in the same group of individuals.^1^ The graph of this study has two distinct peaks that are separated by a period of 4 years. This gap between the peaks may have been too long, and it could have prevented a more precise review, since it lacked the sensitivity to detect minor variations. The information it contains, however, cannot be overlooked. The fact that a single professional assessed the patients and indicated the method of treatment standardises this assessment and reduces the bias of the examiner. By contrast, it reveals just how complex decisions regarding treatment can be. Patients who refuse to undergo surgery may not have such a marked improvement, but the long-term outcome does not differ from that of patients for which surgery was not indicated.

Future studies should include the presence of adverse effects and the complications of drug therapy and surgery. In practice, this is a very important topic to address in conversations with the patients and in decision making. In the literature, however, this topic is scarcely described. In a systemic review and recent meta-analysis, Patel et al.^9^ found that the vast majority of studies do not contain this information, and suggest the use of existing literature on adverse events and clinical judgement to weigh these risks when choosing medical or surgical therapy. In this study, none of these aspects was explored.

## Conclusion

The SNOT-22 scores of patients with CRS with an indication for surgery who did not want to undergo the procedure did not differ from the scores of the clinical treatment group after 4 years of follow-up. The average score showed an improvement, but remained relatively high. This information can help improve the management of patients with CRS.

## Conflicts of interest

The authors declare no conflicts of interest.
